# A New Method for Fibrin-Based Electrospun/Sprayed Scaffold Fabrication

**DOI:** 10.1038/s41598-020-61933-z

**Published:** 2020-03-20

**Authors:** Tamer Al Kayal, Paola Losi, Silvia Pierozzi, Giorgio Soldani

**Affiliations:** 1 Laboratorio di Medicina Rigenerativa, Biomateriali e Terapie Avanzate, Institute of Clinical Physiology, National Research Council, Massa, Italy; 2 Laboratori Archa, Pisa, Italy

**Keywords:** Biotechnology, Materials science, Nanoscience and technology

## Abstract

Fibrin is an optimal scaffold for tissue-engineering applications because it mimics the extracellular matrix. Despite this interesting feature, fibrin gel owns only poor mechanical properties that limit its applications. Different approaches have been used for fibrin electrospinning, however all the methods investigated required washing steps, cross-linking agent treatment or immersion. The aim of this work was to produce a bilayered fibrin/polyurethane scaffold by combination of the electrospun method and the spray, phase-inversion method for the preparation of a fibrin nanostructured layer to be attached onto a poly(ether)urethane microporous support layer. The synthetic layer was obtained by the spray, phase-inversion technique onto a rotating metallic collector, while fibrinogen was processed to obtain a nanofibrous structure by electrospinning. Finally, fibrin polymerization was obtained by thrombin solution spraying onto the electrospun nanofibers. SEM analysis showed the formation of filamentous structure with diameter in the range of μm attached onto the synthetic layer. This scaffold could be applied in soft tissue regeneration such as wound healing or as drug delivery system.

## Introduction

Fibrin is a naturally occurring plasma protein that functions as a major element in the coagulation cascade, contributing to clot formation, cell interaction and wound healing. For these biological properties, fibrin gel was widely investigated as a hemostatic agent in surgery^1^, as a scaffold in tissue-engineering applications^2^, as an angiogenic promoter in vascular graft endothelialisation^3^, and as a drug delivery system in wound healing^4^.

Fibrin is an excellent scaffold for tissue-engineering applications because it mimics the extracellular matrix and improves cellular interaction with the scaffold. Despite these features, the poor mechanical properties of fibrin gel render it unsuitable for clinical applications in which it is necessary to handle fibrin gel or to provide mechanical strength, such as in wound healing. For these reasons, many researchers tried to combine fibrin with synthetic scaffolds^5–7^.

Electrospinning has been successfully employed to obtain nanostructured scaffold fibrin, however all the investigated methods required washing steps for water soluble polymer removal^8^, or thrombin and CaCl_2_ bath^9^ or cross-linking agent use such as glutaraldehyde vapour^10,11^.

The aim of this work was to produce a bilayered fibrin/polyurethane scaffold, by combination of the electrospun method and the spray, phase-inversion method for the preparation of a composite scaffold constituted by a fibrin nanostructured layer attached onto a polyurethane microporous support layer.

## Results

### Fibrin-based electrospun/sprayed scaffold characterization

**Figure 1:**
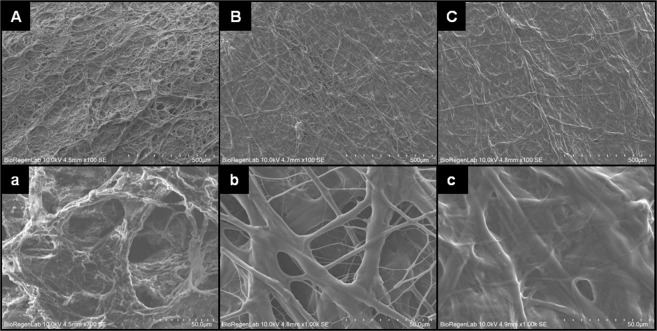
SEM images of bilayered graft: (**A**) and (a) Spray phase inversion microporous layer, (**B**) and (b) Electrospun fibrin fiber layer, (**C**) and (c) Electrospun fibrin layer after thrombin spray treatment. (O.M. 100×, 700× and 1000×).

SEM images of poly(ether)urethane scaffold showed a microporous surface with pores of diameter 41.8 ± 21.5 μm (Fig. 1A,a), while the electrospun fibrinogen on the spray layer loaded with thrombin fibrin fiber resulted in a diameter on the micron order (4.8 ± 3.5 μm) (Fig. 1B,b). The final treatment with thrombin solution, through the spray technique induces a modification of the morphology of the electrospun filament fibrin layer. In particular, SEM images showed a flattened and fused fiber structure with diameter of 4.2 ± 2.9 μm (Fig. 1C,c). The scaffold final thickness was 230 ± 23 µm.

### Peeling test

**Figure 2:**
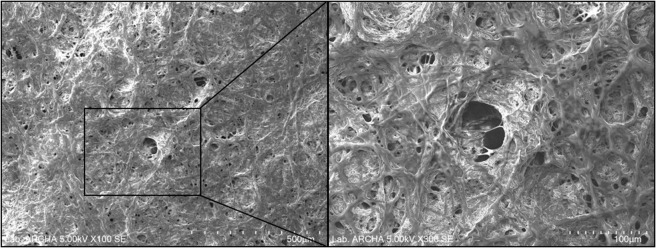
SEM images of poly(ether)urethane layer after peeling of electrospun fibrin layer (O.M. 100× and 300×).

Manual peeling test revealed that the nanostructured fibrin layer was adherent onto the synthetic microporous surface of the bilayered scaffold.The SEM analysis of morphological structure of poly(ether)urethane spray layer after peeling of electrospun fibrin layer, revealed an adhesion of the layer onto the spray surface of the graft indicated by the presence of fused fibrin fiber on the porous synthetic support layer (Fig. 2).

## Discussion

In a previous study we showed that the combination of spray and electrospinning techniques was successfully used for the fabrication of bilayered synthetic graft^12^. The material choice for the fabrication of two different layers, fibrin and polyurethane, was based on our previous experience in manufacturing scaffolds for wound healing applications^4,5^. These studies had shown the capability of fibrin as a delivery system for growth factors, platelet lysate, and of polyurethane as handling support either *in vitro* or *in vivo* experiments.

In this study we demonstrated the possibility of combining two different consolidated techniques to manufacture an electrospun fibrin layer firmly attached onto a polyurethane sprayed layer to enhance fibrin handling with the aim of application in soft tissue regeneration, such as in wound healing application. Moreover, since this manufacturing process does not require washing or immersion steps, it can be useful in the preparation of drug delivery devices.

## Materials and Methods

### Fibrin-based electrospun/sprayed scaffold fabrication

The Fibrin-based scaffold was fabricated by a combined spray/electrospinning apparatus equipped with metallic rotating cylindrical collectors. The support and the microporous layers were obtained using a biocompatible aromatic poly(ether)urethane (Estane 5714F1, Lubrizol, Oevel-Westerlo, Belgium) by a spray, phase-inversion method as previously described^13^. Polyurethane solutions (2 and 0.2%) were prepared by dissolving polyurethane grain in a solvent mixture of tetrahydrofuran and 1,4-dioxane (1:1).The 0.2% solution was brought close to the point of precipitation by the addition of 17% (v/v) of distilled water as a non-solvent. The support layer was obtained by co-spraying the 2% polyurethane solution and distilled water, while the microporous layer by the 0.2% polyurethane solution and thrombin solution at 25 U/mL in 275 mM CaCl_2_ on a collector of 5 cm in diameter at 88 rpm and flow rate of 2 mL/min for both solutions.

Then, a fibrinogen solution for electrospinning was prepared by dissolving the fibrinogen (80 mg/mL) in a solvent mixture of 1,1,1,3,3,3-heaxafluoro-2-propanol and distilled water (9:1, v/v). The fibrinogen layer was electrospun on the poly(ether)urethane microporous layer. Electrospinning was performed using needle-to-collector distance of 10 cm, voltage of 22 kV, flow rate of 0.5 mL/h and rotation speed of 125 rpm. The process was prolonged for 3 h to obtain an about 100 μm thick electrospun network.

Finally, thrombin solution (25 U/mL) was sprayed on the electrospun layer at a flow rate of 0.5 mL/min for 5 min and the scaffolds was incubated at 37 °C for 30 min to allow fibrin polymerization and scaffold drying.

Human fibrinogen and thrombin were supplied by Sigma-Aldrich (S. Louis, MO, USA).

### Fibrin-based electrospun/sprayed scaffold characterization

The structure of the layers of fibrin-based scaffolds was evaluated by a scanning electron microscope (FlexSEM 1000, Hitachi, Tokyo, Japan). Scaffold samples (n = 3) were dry after the electrospinning process; therefore they did not require other treatments before SEM analysis. SEM microphotographs were taken at 100, 300, 700 and 1000x magnifications with a 5 or 10 kV acceleration voltage. The images (n = 5) acquired at 1000x were analyzed by a free, open source image processing program (ImageJ) to quantitatively determine the fibrin nanofibers mean diameter before and after reaction with the thrombin solution. Six random measurements were performed for each image.

### Peeling test

Fibrin layer adhesion onto the synthetic surface of the scaffolds (n = 3) was qualitatively verified by pulling the sample edge with forceps and observing the presence of fibrin nanofibers on the synthetic material. Scaffold sampleswere analyzed by SEM to evaluate the morphological structure of the surface sprayed layer after peeling of electrospun layer^12^.
